# The Future of Bone Repair: Emerging Technologies and Biomaterials in Bone Regeneration

**DOI:** 10.3390/ijms252312766

**Published:** 2024-11-27

**Authors:** Julia Weronika Łuczak, Małgorzata Palusińska, Damian Matak, Damian Pietrzak, Paweł Nakielski, Sławomir Lewicki, Marta Grodzik, Łukasz Szymański

**Affiliations:** 1 Department of Molecular Biology, Institute of Genetics and Animal Biotechnology, Polish Academy of Sciences, Postępu 36A, 05-552 Magdalenka, Poland; j.luczak@igbzpan.pl (J.W.Ł.); m.palusinska@igbzpan.pl (M.P.); 2 Department of Nanobiotechnology, Institute of Biology, Warsaw University of Life Sciences, Ciszewskiego 8, Bldg. 23, 02-786 Warsaw, Poland; marta_grodzik@sggw.edu.pl; 3 European Biomedical Institute, 05-410 Jozefów, Poland; d.matak@ebi.bio; 4 Division of Parasitology and Parasitic Diseases, Department of Preclinical Sciences, Institute of Veterinary Medicine, Warsaw University of Life Sciences, 02-786 Warsaw, Poland; damianpietrzak23@gmail.com; 5 Department of Biosystems and Soft Matter, Institute of Fundamental Technological Research, Polish Academy of Sciences, Pawińskiego 5B, 02-106 Warsaw, Poland; pnakiel@ippt.pan.pl; 6 Institute of Outcomes Research, Maria Sklodowska-Curie Medical Academy, Pl. Żelaznej Bramy 10, 00-136 Warsaw, Poland; slawomir.lewicki@uczelniamedyczna.com.pl

**Keywords:** bone regeneration, fractures, bone grafts, bone substitutes, bone implants

## Abstract

Bone defects and fractures present significant clinical challenges, particularly in orthopedic and maxillofacial applications. While minor bone defects may be capable of healing naturally, those of a critical size necessitate intervention through the use of implants or grafts. The utilization of traditional methodologies, encompassing autografts and allografts, is constrained by several factors. These include the potential for donor site morbidity, the restricted availability of suitable donors, and the possibility of immune rejection. This has prompted extensive research in the field of bone tissue engineering to develop advanced synthetic and bio-derived materials that can support bone regeneration. The optimal bone substitute must achieve a balance between biocompatibility, bioresorbability, osteoconductivity, and osteoinductivity while simultaneously providing mechanical support during the healing process. Recent innovations include the utilization of three-dimensional printing, nanotechnology, and bioactive coatings to create scaffolds that mimic the structure of natural bone and enhance cell proliferation and differentiation. Notwithstanding the advancements above, challenges remain in optimizing the controlled release of growth factors and adapting materials to various clinical contexts. This review provides a comprehensive overview of the current advancements in bone substitute materials, focusing on their biological mechanisms, design considerations, and clinical applications. It explores the role of emerging technologies, such as additive manufacturing and stem cell-based therapies, in advancing the field. Future research highlights the need for multidisciplinary collaboration and rigorous testing to develop advanced bone graft substitutes, improving outcomes and quality of life for patients with complex defects.

## 1. Introduction

Bone defects and fractures resulting from trauma, degenerative diseases, congenital abnormalities, or tumor resections present significant clinical challenges in the fields of orthopedic and maxillofacial surgery. While natural bone has a remarkable ability to heal minor defects, large or critical-sized defects often require the use of bone implants or grafts to restore functionality and structural integrity. Moreover, delayed or failed healing, which can affect up to 10% of fractures, may result from several factors, including comminuted fractures, infection, tumors, or compromised vascular supply. The process of bone fracture healing is complex and multifaceted. Consequently, any disruption to this process can significantly reduce a patient’s quality of life. Given that 5–10% of fracture cases result in delayed healing or non-union, this issue represents a significant global public health challenge that demands immediate and targeted intervention [1,2,3]. In response to this need, the field of bone tissue engineering has evolved rapidly over the past few decades, driven by the growing demand for durable, biocompatible, and bioactive materials capable of supporting and enhancing bone regeneration. The ideal bone implant must satisfy several stringent criteria, including biocompatibility, osteoconductivity, osteoinductivity, and the ability to integrate seamlessly with surrounding tissues. Traditionally, autografts and allografts have been the gold standard for bone replacement. However, the limitations of these solutions, such as limited availability, donor site morbidity, immune rejection, and risk of disease transmission, have prompted the development of alternative synthetic and bio-derived materials. Recent advances in biomaterials science, 3D printing technologies, and regenerative medicine have led to the development of a new generation of bone implants. These implants aim to replace the damaged or missing bone but also stimulate the body’s natural bone-healing processes [4]. Materials such as metals (titanium and its alloys), ceramics (hydroxyapatite, calcium phosphate), polymers (biodegradable and non-biodegradable), and composite materials have all been investigated for their potential in bone tissue engineering. Moreover, bioactive coatings and surface modifications have been employed to enhance the osteointegration of these materials [5]. In addition to conventional approaches, photobiomodulation therapy (PBMT) has emerged as a promising adjunctive technique for enhancing bone repair. PBMT operates by stimulating cellular metabolism and increasing protein synthesis, thereby facilitating bone regeneration [6]. Clinically, PBMT has demonstrated potential in augmenting bone formation, promoting osteoblast differentiation, and enhancing protein deposition, collectively contributing to the development of new bone tissue [7]. While PBMT is utilized across various disciplines, including nursing, dentistry, and physical therapy [8], challenges persist in the standardization of treatment protocols and a comprehensive understanding of its mechanisms and long-term effects on bone functionality [9]. Future advancements in PBMT may harness innovative technologies, such as biocompatible optical fibers and minimally invasive needle systems, to optimize its efficacy and broaden its clinical applications [10]. This review seeks to provide a comprehensive overview of the current state of bone implant technologies, focusing on the latest advancements in materials, design, and clinical applications. It will explore the biological mechanisms underlying bone regeneration, the challenges faced in creating optimal bone implants, and the future directions of research in this dynamic and interdisciplinary field. In particular, we will examine the role of emerging technologies such as additive manufacturing, biofunctionalization, and the use of growth factors and stem cells in improving the performance of bone implants. By synthesizing the current body of knowledge, this review aims to offer insights into the evolving landscape of bone implant research and highlight promising strategies for developing next-generation implants that meet clinical needs and push the boundaries of regenerative medicine.

## 2. Epidemiology and Causes of Bone Fractures

Age is one of the most critical factors in bone union disorders, particularly as the global population ages. By 2050, the elderly population is projected to reach 21.1% globally, with notable increases in countries like the United States, where the number of individuals over 65 is expected to double to 83.7 million [11]. This demographic shift will likely lead to a significant rise in bone fractures, especially among older adults prone to osteoporosis and reduced bone density [12]. Young individuals often sustain fractures from high-energy trauma, such as sports injuries or road accidents, while age-related conditions like osteoporosis compound fracture risk in the elderly [11]. Road accidents alone injure millions annually, adding to the burden on healthcare systems. The prevalence of osteoporosis further exacerbates the fracture incidence, with 10 million Americans over 50 affected, leading to 1.5 million fractures yearly [13]. In 2010, 22.1% of women and 6.6% of men over 50 in the EU had osteoporosis, contributing to 3.5 million fractures [14].

Several clinical risk factors contribute to fracture risk independently of bone mineral density (BMD), including age, prior fragility fractures, smoking, excess alcohol consumption, family history of hip fractures, rheumatoid arthritis, and the use of oral glucocorticoids [15]. Genetic factors also play a crucial role in bone formation and skeletal fragility, as shown in twin and family studies that indicate the high heritability of BMD, primarily affecting peak bone mineral mass acquisition rather than age-related bone loss. This underscores the importance of early-life interventions [16]. Environmental pollutants, such as heavy metals, air pollutants, endocrine disruptors, metal ions, and trace elements, further exacerbate the risk of osteoporosis and bone fractures by disrupting normal bone metabolism [17]. External factors like dietary habits, exercise, menstrual irregularities, medications, underlying disease states, body weight, and environmental conditions significantly impact fracture risk as well [18].

Notably, fall-related and bone-related risk factors consistently persist regardless of fracture location, patient age, or gender, indicating that integrated assessments of bone health and fall risk are essential for identifying elderly individuals at the highest fracture risk. Lower serum estradiol levels, elevated homocysteine, and the use of certain antihypertensive medications have also been linked to increased fracture risk [19]. Collectively, these risk factors underscore the growing need for comprehensive healthcare resources to address the anticipated rise in fracture-related treatments worldwide.

## 3. Bone Regeneration

**Figure 1:**
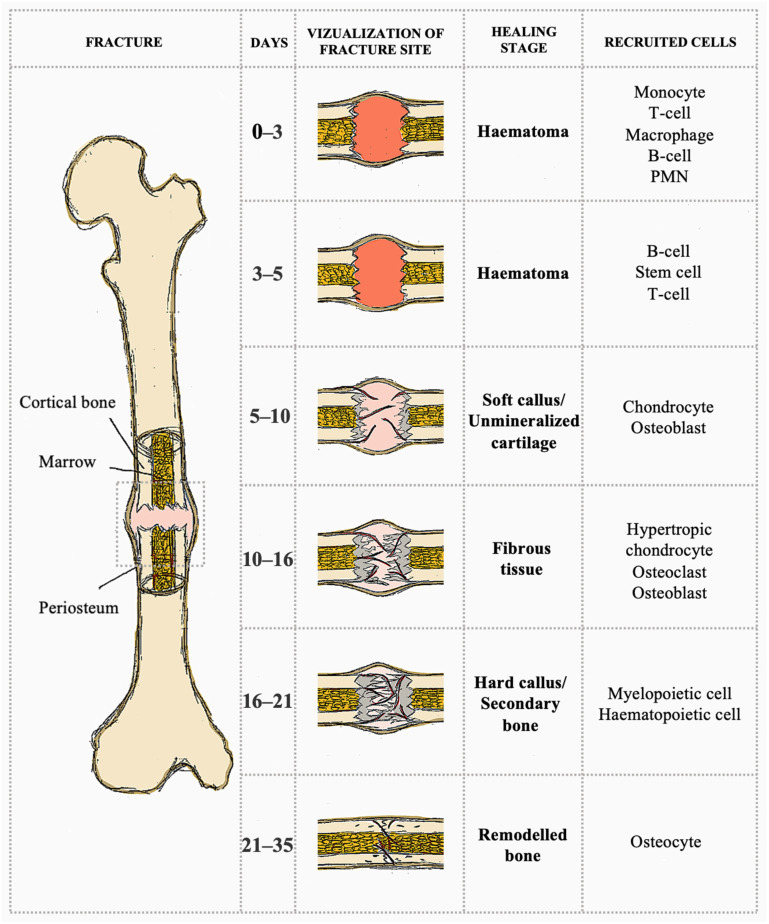
Typical process of fracture healing, highlighting the biological events and cellular activities occurring at each stage. Abbreviation: PMN refers to polymorphonuclear leukocytes. (Based on: [20]). Created with GIMP 2.10.38.

Bone fracture healing is a complex and dynamic regenerative process to restore the damaged bone to its original condition and cellular structure [20]. A fracture represents a disruption in the bone cortex’s structural continuity, often accompanied by varying degrees of damage to the surrounding soft tissues. The healing process commences with secondary healing, which unravels in four stages: the formation of a hematoma, the development of granulation tissue, the formation of a bony callus, and bone remodeling (Figure 1). Principal cells and their secretions are integral to the healing process, with mesenchymal stem cells (MSCs) serving a pivotal role [21]. The type of fracture healing depends on the mechanical stability achieved at the fracture site and the corresponding strain levels. Mechanical stimulation, such as strain, is of significant importance in the promotion of tissue formation at the ends of the fractured bone. The degree of strain directly influences the cellular activities engaged in the healing process, which in turn determines the type of bone healing that occurs [22]. Bone healing can be divided into two principal categories. Primary bone healing, which is governed by constructs that achieve absolute stability and maintain mechanical strain below 2%, involves intramembranous bone healing through Haversian remodeling. Secondary bone healing, the most prevalent form of bone healing, occurs when there is a minimal amount of motion at the fracture site. The interfragmentary motion results in the formation of a soft callus, which subsequently gives rise to secondary bone formation through both intramembranous and endochondral ossification [23]. This type of healing is characterized by endochondral ossification. In certain instances, the process of bone healing may entail a combination of primary and secondary mechanisms, contingent upon the stability of the fixation throughout the healing period [24].

Bone regeneration is a complex, tightly regulated process orchestrated by various endogenous factors that ensure the repair and remodeling of damaged bone tissue. These factors include signaling molecules, growth factors, and cellular components that work together to restore bone integrity [25]. Among the most critical endogenous factors are bone morphogenetic proteins (BMPs), a group of growth factors belonging to the transforming growth factor-beta (TGF-β) superfamily, with the exception of BMP1, which is a metalloprotease. BMPs, particularly BMP-2 and BMP-7, play pivotal roles in initiating the differentiation of MSCs into osteoblasts, promoting bone formation. Other key growth factors include vascular endothelial growth factor (VEGF), which stimulates angiogenesis to ensure adequate blood supply during bone repair [26,27], and insulin-like growth factors (IGFs), which support osteoblast proliferation and matrix production [28]. In addition to growth factors, cytokines such as interleukins (ILs) and tumor necrosis factor-alpha (TNF-α) are involved in modulating inflammation during the early stages of bone healing, setting the stage for tissue regeneration [29,30,31]. The Wnt/β-catenin signaling pathway is also crucial, as it regulates osteoblasts by inducing the expression of osterix, a transcription factor crucial for osteoblast maturation, and osteoprotegerin, which inhibits osteoclast formation, thereby balancing bone mass homeostasis [32,33]. The significance of this signaling pathway is thoroughly elucidated by Hu et al. (2024) [34]. It is noteworthy that the Wnt/β-catenin signaling pathway has the capacity to stimulate the proliferation and migration of cholangiocarcinoma cells, a process that involves the involvement and action of MSCs and MSCs-CM. This underscores the broader impact of this signaling pathway on cell proliferation and migration [35]. The RANK/RANKL/OPG axis is another essential system that governs the balance between bone resorption and formation by regulating osteoclast activity [36]. RANKL (Receptor Activator of Nuclear Factor Kappa-B Ligand) binds to its receptor RANK (Receptor Activator of Nuclear Factor Kappa-B), which facilitates osteoclast differentiation and activation, ultimately resulting in increased bone resorption. Conversely, OPG (Osteoprotegerin) functions as a decoy receptor that binds to RANKL, thereby preventing its interaction with RANK. This inhibition of RANKL–RANK signaling effectively suppresses osteoclastogenesis and promotes bone formation [37,38,39]. Endogenous stem cells, particularly MSCs, contribute significantly to bone regeneration by differentiating into osteoblasts under the influence of these signaling factors [40]. Together, these endogenous factors orchestrate the sequential phases of bone regeneration, ensuring the restoration of bone structure and function.

## 4. Composition of Native Bone and Properties of Ideal Substitutes

The development of an ideal bone substitute is a complex challenge that requires careful consideration of multiple factors. Such a substitute must be biocompatible, bioresorbable, and osteoconductive, ensuring that it can serve as a scaffold for bone healing while simultaneously allowing for new bone formation. It should maintain these properties until the bone is sufficiently healed and the substitute is no longer needed [24,41]. Additionally, the bone substitute should possess osteogenic, osteoinductive, and osteoconductive properties to actively stimulate bone formation and support the healing process [42,43,44]. To enhance regenerative properties, the optimization of controlled growth factor release is crucial. This requires the design of biomaterial scaffolds that balance release kinetics to sustain tissue repair while preserving bioactivity [45]. Such optimization is often facilitated by mechanisms involving biodegradable polymers and nanoparticle carriers [46]. Additionally, active control of implants using external stimuli, such as electromagnetic fields (EMF) or PBMT, can be employed to trigger the targeted release of therapeutic molecules, thereby enhancing bone regeneration and healing [47,48,49,50]. From a mechanical perspective, it is crucial that the bone substitute provides satisfactory support and stability. It should exhibit bone-like mechanical properties and maintain biomechanical stability to withstand the local load environment specific to the application [51,52]. This balance between biological and mechanical requirements is particularly challenging, especially when developing substitutes for load-bearing applications in bone repair. Achieving optimal biocompatibility and osseointegration necessitates that the bone substitute has a hydrophilic nature, an effective interface with human bone, and handles well under clinical conditions. Moreover, it must be easy to sterilize to ensure safety during surgical procedures. Given these complexities, extensive preclinical experimentation and the standardization of studies are essential for the reliable evaluation of biological bone substitute materials, particularly in highly loaded skeletal sites [53]. The selection of an optimal bone graft must, therefore, involve a comprehensive assessment of biomechanical, biomaterial, biological, and clinical considerations to achieve the best possible clinical outcome and patient satisfaction [54,55].

## 5. Current Solutions

**Figure 2:**
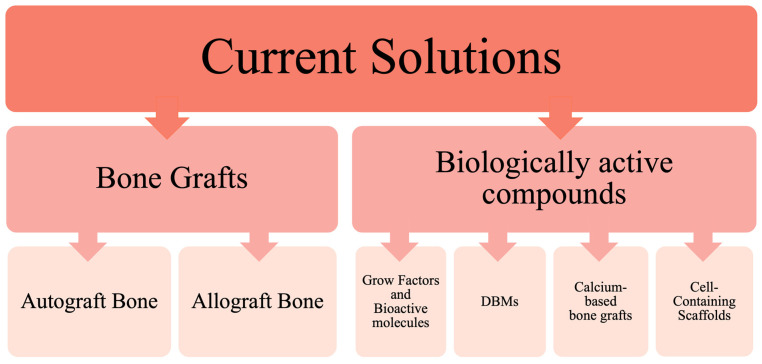
Classification scheme dividing bone grafts and their substitutes into main groups based on the basic material composition and processing method. BMP, Bone morphogenic protein; PDGF, Platelet-derived growth factor; DBM, Demineralized bone matrix. Created with GIMP 2.10.38.

Bone healing following trauma or orthopedic surgery does not always progress as expected. Despite advancements in internal bone fixation techniques, including titanium plates and nails, factors such as insufficient vascularization and significant loss of bone or soft tissue can hinder effective bone regeneration. Consequently, the demand for bone substitutes and growth stimulators has risen sharply. Currently, a diverse array of bone graft substitutes is available, classified into six primary categories based on material composition and processing methods, as illustrated in Figure 2.

The current gold standard for repairing bone defects remains using autologous bone grafts, where bone is sourced directly from the patient [56,57]. These grafts are naturally compatible with the patient’s body, eliminating the risk of immune rejection and providing the necessary biological properties for successful bone grafting. Autografts inherently possess the critical elements needed for osteoinduction, osteogenesis, and osteoconduction, making them highly effective in bone healing [58]. The main source of autologous bone for grafting is the iliac crest due to its accessibility and the abundance of progenitor cells and growth factors [59]. However, obtaining autografts necessitates an additional surgical procedure at the donor site, which can lead to complications such as injury, morbidity, deformity, and scarring. The process of harvesting and implanting these grafts is not only costly but also carries significant surgical risks, including bleeding, inflammation, infection, chronic pain, and other postoperative issues [60,61,62]. Moreover, autografts may be unsuitable when the bone defect is extensive and requires a large volume of bone [63,64]. In cranio-maxillofacial surgeries, the use of autografts has declined, with allografts becoming more prevalent in the United States and bovine xenografts being preferred in Europe due to cost-effectiveness and other practical considerations [65].

## 6. Growth Factors and Bioactive Molecules in Bone Repair

The growing necessity for efficacious treatments of impaired bone tissue has prompted the pursuit of sophisticated techniques in bone tissue regeneration. Growth factors and bioactive peptides are of pivotal importance in the field of bone tissue engineering [66]. The use of hormonal signals to harness osteogenic potential is particularly relevant in the context of addressing osteoporosis-related bone defects. In light of the inherent instability of protein factors and the non-specific actions of hormones, the design of bone substitutes for the controlled release of these bioactive agents is of paramount importance.

### 6.1. Bone Morphogenic Proteins–BMPs

BMPs are multifunctional growth factors that belong to the TGF-β superfamily. They are renowned for their ability to enhance bone grafts and play a pivotal role in the healing of fractures and non-union bones [67]. BMPs play crucial roles in the processes of chemotaxis, mitogenesis, and the differentiation of MSCs, as well as in promoting angiogenesis [68,69,70,71]. Different BMPs are expressed during bone formation, following distinct spatial and temporal patterns, and each has specific functions during in vivo bone morphogenesis [72]. The most widely recognized BMPs influencing the regenerative process are BMP-2 and BMP-7.

BMP-2 is increasingly utilized in orthopedic surgery, including dental procedures, open tibial fractures, cancer treatment, and spinal surgery, due to its association with rapid healing and minimal risk of rejection or infection. BMP-2 induces the differentiation of osteoprogenitor cells into osteoblasts and stimulates the formation of new bone, which is critical in spinal fusion surgery and the treatment of nonunion in long bone fractures [73]. However, off-label use has resulted in various adverse effects, prompting ongoing research aimed at optimizing treatment by refining the concentration, dosage, carrier type, and delivery methods [74]. Lim and colleagues (2021) evaluated the bone regenerative capacity of different biphasic ceramic scaffold types combined with recombinant human bone morphogenetic protein-2 (rhBMP-2) at varying concentrations in a rabbit model [75]. The results demonstrated that the addition of rhBMP-2 significantly enhanced bone formation, particularly in the early stages of regeneration. Importantly, the findings showed that higher concentrations of rhBMP-2 did not correlate with increased bone regeneration, indicating that there is a threshold for its efficacy. In a separate study, Raina et al. (2020) developed and characterized a gelatin-nanohydroxyapatite (GM)-based bone bandage designed for the controlled delivery of rhBMP-2 and zoledronic acid (ZA), specifically aimed at treating fracture nonunions [76]. The GM scaffold exhibited excellent biocompatibility, supporting osteogenic differentiation of MC3T3 cells, and maintained rhBMP-2 bioactivity over a 5-week release period. In vivo analysis in a rat model revealed that the GM + rhBMP-2 + ZA group exhibited significantly enhanced bone volume (21.5 ± 5.9 mm^3^ vs. 2.7 ± 1.0 mm^3^) and bone area (3.3 ± 2.3 mm^2^ vs. 1.0 ± 0.4 mm^2^) relative to the GM + rhBMP-2 group after four weeks. Furthermore, in a rat nonunion model, GM functionalized with rhBMP-2 + ZA achieved more significant bone formation (63.9 ± 19.0 mm^3^ vs. 31.8 ± 3.7 mm^3^) and increased fracture callus strength (110.8 ± 46.8 N vs. 45.6 ± 17.8 N) compared to untreated controls. Despite these significant enhancements in bone regeneration and biomechanical properties, the overall union rate was only marginally improved. Additionally, GM alone or combined with ZA did not significantly promote bone healing in this model. In an alternative approach, Dai et al. (2020) developed absorbable gelatin scaffolds loaded with rhBMP-2 and evaluated their ability to induce vascularized juvenile ossicle formation in aged mice [77]. These scaffolds significantly promoted ossicle generation, requiring fewer mesenchymal stem cells and exhibiting a higher density of type H blood vessels than native bone, underscoring their regenerative potential. In vivo experiments further demonstrated that these rhBMP-2-induced ossicles enhanced bone repair in critical-sized cranial defects in both young and aged mice.

One of the most well-known products containing rhBMP-2 is Infuse^™^ Bone Graft (Medtronic, Minneapolis, MN, USA) [78]. The product has been approved by the Food and Drug Administration (FDA) for numerous applications in bone regeneration, including anterior lumbar interbody fusion (2002), tibial nonunion (2004), and as an alternative to autogenous bone graft for sinus augmentations and localized alveolar ridge augmentations for defects associated with extraction sockets (2007). The operational principle is the utilization of an rhBMP-2 formulation applied to an absorbable collagen sponge carrier (ACS), whereby one of the functions of the protein is to stimulate the formation of new bone tissue in the body [79]. Medtronic has tried to introduce an additional product based on rhBMP-2, branded as Amplify^®^. However, this product remains unavailable in the marketplace due to numerous controversies. Several studies have raised questions about its efficacy and safety, leading to significant debate within the scientific community and media [80,81]. Despite numerous documented instances of successful bone regeneration utilizing this product [82], the use of this graft type, as previously stated, has been linked to complications, particularly in instances where the product is used in ways that do not follow its approved indications. The FDA has issued a caution letter regarding the use of Infuse off-label in anterior cervical fusions, citing the potential for massive soft-tissue swelling, which could compromise a patient’s airway [83,84].

Another BMP of particular significance within this family is BMP-7, which possesses the ability to induce bone formation and has been used clinically to promote vertebral fusions and the healing of non-union fractures [85]. Studies conducted by Feng et al. (2012) demonstrated that BMP-7, when combined with various scaffolds like chitosan/nHAC composites, significantly accelerates bone regeneration in cranial bone defects in rats [86]. Additionally, BMP-7 enhances osteogenic differentiation and mineralization of human bone marrow-derived stem cells cultured on bovine bone particles [87]. A number of clinical trials have been conducted to evaluate the safety and efficacy of recombinant BMP-containing devices. The findings of these trials have informed the development of several final products currently available on the market. A product containing rhBMP-7 was initially approved in 2001 as an alternative to autograft for treating persistent long bone nonunions when autografts and other treatments were not viable [88]. It subsequently received approval for use in revision posterolateral lumbar fusion in 2004 [89]. The rhBMP-7 graft, bound to a collagen carrier, was available for a limited period before being withdrawn from the global market [90].

### 6.2. Bioactive Peptides

Growth factor-based therapies frequently entail considerable expense and may result in unfavorable outcomes, including immune responses in certain patients. To address these challenges, a range of bioactive peptides have been investigated as potential alternatives to traditional growth factors. Bioactive peptides are short chains of amino acids (typically 2–20 residues) derived from proteins that exhibit various beneficial biological activities, typically derived from the functional domains of proteins [91]. These analogs of biologically active proteins can exert a significant influence on several key biological and physiological processes, regulate cellular functions, and enhance intercellular communication [92,93,94]. The production of these peptides is cost-effective due to their small size, which facilitates straightforward design and synthesis, and their reduced risk of immunogenicity [95]. Bioactive peptides can be derived from a number of sources, including extracellular matrix (ECM) proteins such as collagen, fibronectin, bone sialoprotein, and laminin, as well as soluble growth factors, anabolic protein hormones, and both engineered and naturally occurring peptides [96]. A significant number of bioactive peptides have demonstrated considerable potential in the promotion of bone formation in both in vitro and in vivo environments [97,98,99,100]. Bioactive peptides can be classified into three functional categories: those that enhance osteo-differentiation, those that facilitate cell adhesion, and those that contribute to neovascularization, as presented in Table 1. Many of these peptides have been employed in clinical settings for tissue engineering purposes. Of these, Parathyroid hormone 1-34 (PTH1–34) and P-15 have received FDA approval for therapeutic use, while thrombin peptide 508 (TP508) has been evaluated in phase I/II clinical trials.

Among these, iFactor (Cerapedics, Broomfield, CO, USA), also known as Anorganic Bone Matrix (ABM) combined with Peptide 15 (P-15), has emerged as a notable contender in the field of bone regeneration. This bone graft combines the P-15 Osteogenic Cell Binding Peptide with an anorganic bone mineral (ABM), enabling a unique “attract, attach, activate” mechanism that enhances the body’s natural bone healing process while minimizing the risk of ectopic bone growth. It is terminally sterilized, can be stored at room temperature, and boasts a shelf life of three years. Clinical studies have shown that P-15 is safe and improves outcomes in various applications [101,102,103]. Mobbs et al. conducted a prospective study on patients with degenerative spinal disease using the iFactor Bone Graft [104] among 110 patients undergoing single or multilevel anterior lumbar interbody fusion (ALIF); the mean follow-up was 24 months. Radiographic evidence of bony induction was observed in all patients, with fusion rates of 97.5%, 81%, and 100% for single-, double-, and triple-level surgeries, respectively. Similarly, Arnold et al. reported on a cohort of 220 participants from the US FDA IDE trial for single-level anterior cervical discectomy and fusion (ACDF) [105]. After 72 months, fusion rates were 99.0% for iFactor Bone Graft and 98.2% for autograft, with minimal differences in Neck Disability Index improvement, neurological success, and overall success rates. Both groups had comparable pain and SF-36 scores with no adverse effects. The iFactor Bone Graft showed similar results to autografts in ACDF and demonstrated promising outcomes in ALIF surgeries for degenerative spinal conditions.

TP508 is a 23-amino-acid peptide that represents a portion of the receptor-binding domain of human thrombin, a naturally occurring molecule in the body that is responsible for blood clotting and for initiating numerous cellular events involved in tissue repair [106]. The administration of TP508 has been demonstrated to accelerate bone formation and consolidation during distraction osteogenesis in animal models [107,108]. It facilitates bone regeneration by modulating the expression levels of proteins associated with the cell cycle, cellular growth, and proliferation, thereby promoting cell growth through the regulation of cell survival signals over cell death signals [109]. TP508 may prove to be a valuable adjunct in cases requiring augmentative treatment for bone formation and consolidation. It has been demonstrated to enhance bone healing and mitigate the risk of complications such as muscle fibrosis and delayed or non-union fractures in high-energy fracture conditions [110]. Furthermore, TP508 has demonstrated efficacy in promoting the healing of both critically and non-critically sized segmental bone defects [111]. Nevertheless, despite these encouraging outcomes, the precise molecular mechanisms and specific pathways by which TP508 induces osteoblast proliferation and differentiation remain incompletely understood. Further research is required to evaluate the long-term effects of TP508 in implant-based rehabilitation and to elucidate the underlying processes that regulate osteoprogenitor cellular responses.

PTH1-34, also known as teriparatide, is a recombinant form of N-terminally truncated and 34-amino acid-containing human parathyroid hormone (1–34). The pharmaceutical company Eli Lilly (Toronto, ON, Canada) markets the drug under the brand name Forteo. It is indicated for use as an anabolic agent in postmenopausal women with osteoporosis [112]. PTH1-34 has been demonstrated to facilitate bone formation by influencing osteoinduced adipose-derived stem cells (ADSCs) through the modulation of SIK2 and Wnt4 signaling pathways. Specifically, PTH1-34 has been demonstrated to phosphorylate SIK2, upregulate RANKL, and downregulate SOST, thereby enhancing the osteogenesis process of ADSCs [113]. The knockdown of Wnt4 has been demonstrated to influence the expression of downstream osteogenic proteins, thereby inhibiting the osteogenic differentiation of ADSCs. Moreover, PTH1-34 has been demonstrated to enhance the autophagic activity of osteoblast precursors, a process that is integral to PTH1-34-mediated osteoblast formation [114]. PTH1-34 has been demonstrated to stimulate osteoblast formation and activate autophagy, which plays a pivotal role in osteoblastogenesis. In vivo studies have demonstrated that PTH1-34 not only improves bone loss but also restores the autophagic activity of immature osteoblasts within bone tissues. Furthermore, it enhances autophagy in osteoblast precursors, thereby promoting osteogenic differentiation and mineralization via the cAMP/PKA signaling pathway [115]. PTH1-34 has been used in clinical practice to increase bone mass, reduce the risk of fractures in postmenopausal women with osteoporosis, and facilitate fracture healing. Preclinical studies have demonstrated that PTH1-34 accelerates callus formation, enhances bone remodeling, and improves the biomechanical properties of the healing fracture. These findings suggest that PTH1-34 may have potential clinical applications for promoting fracture union in cases of osteoporosis and non-unions [116]. The use of PTH1-34 should be followed by potent antiresorptive therapy to sustain gains in BMD and bone strength [117]. However, long-term administration of PTH1-34 has been associated with de novo osteoarthritis and bone deformation, emphasizing the necessity for close patient monitoring during therapy [118].

### 6.3. Platelet-Derived Growth Factor

Platelet-derived growth factor (PDGF) is a principal mitogen for connective tissue cells, performing crucial functions in embryonic development, wound healing, and a multitude of pathological conditions [119]. PDGF has been demonstrated to promote mitogenesis, angiogenesis, and macrophage activation, thereby facilitating bone regeneration [120]. In the context of normal fracture repair, the A-chain of PDGF is expressed by a range of cell types, including endothelial cells, mesenchymal cells, osteoblasts, chondrocytes, and osteoclasts. In contrast, the B-chain of PDGF is primarily observed in osteoblasts during bone formation [121]. PDGF-BB, a specific isoform, functions as a potent chemotactic factor for mesenchymal cells in the context of skeletal tissue repair. It has been demonstrated to promote the infiltration of mesenchymal progenitor cells, as well as chondrogenic and osteogenic responses, and the remodeling of repair tissues in injured growth plates [122]. An understanding of the molecular mechanisms of PDGF in bone regeneration offers potential avenues for enhancing bone repair and addressing conditions such as osteosarcoma [120]. The use of recombinant human platelet-derived growth factor-BB (rhPDGF-BB) in bone graft substitutes has been the subject of extensive investigation with a view to determining its potential to enhance bone regeneration and repair. A substantial body of evidence from both clinical and non-clinical studies has demonstrated that rhPDGF-BB is safe, with no significant toxicity, carcinogenicity, or tumor promotion observed even at high doses. Moreover, clinical trials have demonstrated that products containing rhPDGF-BB do not elevate the incidence of adverse events or cancer risk [123]. rhPDGF-BB has been shown to facilitate bone regeneration when combined with a range of bone graft materials. The combination of rhPDGF-BB with beta-tricalcium phosphate (β-TCP) or deproteinized bovine bone mineral (DBBM) has been demonstrated to significantly enhance bone formation in animal models [124]. Nevertheless, some studies have indicated that the addition of rhPDGF-BB to TCP or DBBM in rodent models did not result in a notable impact on bone formation [125]. In human studies, rhPDGF-BB combined with β-TCP/HA has been demonstrated to be as effective as autogenous bone grafts for guided bone regeneration (GBR) in alveolar ridge augmentation [126]. Furthermore, rhPDGF-BB has shown promising results in the treatment of severe intrabony periodontal defects when combined with xenogeneic bone substitutes, resulting in significant clinical improvements [127]. At present, a restricted range of products have been approved by the FDA for use in bone regeneration processes involving the use of rhPDGF-BB. Such products include specialized bone grafts for orthopedic and dental applications, which are designed to enhance bone repair in specific clinical scenarios. Augment Bone Graft (Stryker, Singapore), a blend of rhPDGF-BB and β-TCP, received FDA approval in 2015 for use in surgical procedures involving tibiotalar joint and hindfoot (including the subtalar, talonavicular, and calcaneocuboid joints) fusion [123]. Additionally, other rhPDGF-BB-based products are available on the market, particularly for dental applications. One example is GEM 21S (Lynch Biologics, Franklin, TN, USA), a bone graft that also combines rhPDGF-BB with β-TCP. This product was approved by the FDA in 2005, specifically for treating various periodontal defects [128].

## 7. Demineralized Bone Matrix

Demineralized bone matrix (DBM) is derived from human allograft bone that has undergone a demineralization process to remove its mineral content [129]. This process typically entails the utilization of acids, such as hydrochloric acid or ethylenediaminetetraacetic acid (EDTA), to facilitate the dissolution of the mineral components, predominantly calcium, from the bone. The extent of demineralization can vary, affecting the residual calcium content and the exposure of bioactive molecules. For example, a residual calcium content of less than 4% is optimal for the exposure of bone collagen fibers and bioactive molecules [130]. Consequently, the matrix retains a substantial concentration of collagens (mainly type I with some types IV and X) and non-collagenous proteins, as well as a diverse range of growth factors, including BMPs, which are vital for promoting bone regeneration [131]. The preservation of these proteins endows DBM with osteoinductive properties, rendering it a valuable tool in facilitating new bone formation across a range of clinical applications. Specifically, BMPs and other growth factors in DBM are known for their potent osteogenic effects, making DBM a valuable tool in promoting bone regeneration and accelerating the healing of bone defects [132,133]. One of the key advantages of DBM is its dual role as both an osteoinductive and osteoconductive material. DBMs have the ability to recruit and stimulate progenitor cells to differentiate into bone-forming cells, thereby initiating bone formation as well as providing a structure that supports the ingrowth of new bone from the surrounding tissue [134,135,136]. Despite these benefits, the clinical use of DBM is not without challenges. The quality and effectiveness of DBM can vary significantly depending on factors like the age and health of the bone donor and the specific techniques used in its processing [137,138,139]. Additionally, DBM often requires a carrier or binding agent to enhance its handling and ease of application during surgery. Without a suitable carrier, DBM can be difficult to manipulate, which may limit its effectiveness in certain procedures. DBM lacks intrinsic mechanical strength; it is frequently employed in conjunction with other materials that provide structural support. For instance, DBM can be employed to augment cortical grafts, thereby enhancing their connectivity and integration with host bone, which is of paramount importance for weight-bearing applications. DBM is utilized in a plethora of orthopedic procedures, including spinal fusions, where it serves as a bone graft enhancer. Nevertheless, it does not offer structural support on its own and is typically deployed to fill bone defects and cavities [140,141]. Further challenges include the relatively limited osteoinductive capacity of DBM when compared to other grafting materials like autografts, which are considered the gold standard due to their high success rates in bone regeneration. Efforts are ongoing to enhance the osteoinductive properties of DBM through improved processing techniques and combination with other biomaterials to ensure more consistent outcomes. This requirement arises from several inherent limitations of DBM, including its challenging manipulation, propensity to migrate from graft sites, and inadequate stability following implantation. Consequently, various carriers have been developed to address these limitations, each offering specific advantages. For example, poloxamer 407-based hydrogel enhances the osteoinductivity of DBM relative to sterile water, thereby promoting improved bone regeneration [142]. Additionally, thermogelling chitosan forms a gel-like composite at physiological temperatures, which significantly enhances handling and stability during surgical procedures [143]. Sodium alginate, when combined with DBM, produces an injectable putty with favorable histocompatibility and osteoinductive properties [144]. Moreover, gelatin methacryloyl (GelMA) demonstrates superior compressive strength, serum cohesivity, and osteoinductive potential compared to other carriers such as glycerol and hyaluronic acid [145]. Similarly, silk fibroin contributes to the formation of a stable composite that minimizes DBM migration while supporting cell attachment and proliferation [146]. In addition, the combination of bovine collagen and alginate has shown significant improvements in clinical outcomes, such as enhanced clinical attachment levels and bone fill in periodontal defect treatments [147]. While the selection of a carrier can influence clinical outcomes, including fusion rates and bone repair capabilities, some studies report that these differences are not always statistically significant [148,149]. DBM products are available in a range of forms, including sponges, strips, injectable putty, paste, and paste infused with chips [150]. The diverse forms of DBM impact its capacity to function as graft extenders, enhancers, or substitutes. This renders DBM a highly adaptable choice for a multitude of bone grafting applications, including periodontal procedures, craniofacial defect repairs, and orthopedic surgeries, where both osteoinduction and osteoconduction are essential for optimal outcomes.

Building on these advancements, several clinical trials have evaluated the efficacy of DBM products in the treatment of non-union fractures and the promotion of bone healing. Notably, the prospective registry and retrospective data collection study (NCT04299022) will evaluate the use of ViviGen Cellular Bone Matrix in patients with acute, delayed, or non-union fractures, as well as in fusion procedures [151]. This study will monitor patients at multiple time points, including hospital discharge, 6 weeks, 3 months, 6 months, 12 months, and 24 months, with outcomes focused on radiographic fracture healing and patient-reported outcomes. Secondary outcomes will include the rate of secondary interventions within twelve months following definitive wound closure. While this and other studies exploring bone matrix products will demonstrate promising results, challenges will remain in standardizing treatment protocols and fully understanding the long-term effects of these materials across diverse fracture types and patient populations.

## 8. Calcium-Based Bone Graft Substitutes

Calcium-based bone graft substitutes, which are known for their biodegradability and bioactivity, represent an innovative solution for enhancing bone regeneration. By capitalizing on the intrinsic characteristics of calcium and phosphate, these substitutes enable a more efficacious integration with the adjacent bone tissue, thereby promoting enhanced healing outcomes [152]. Calcium-based bone grafts are available in a variety of forms, including hydroxyapatite (HA), β-TCP, calcium sulfate, calcium phosphate cements, and composite grafts. Table 2 presents an overview of calcium-based products, which exhibit distinct advantages for specific clinical applications. Each of these has distinct advantages for specific clinical applications.

Among these, HA is one of the most extensively studied and utilized materials due to its favorable biocompatibility and structural similarity to natural bone. HA is a bioactive and biocompatible ceramic material, predominantly composed of calcium and phosphate, which closely mimics the mineral structure of human bones and teeth [170]. HA can be synthesized from natural sources, including coral and eggshells, or produced through synthetic methods [171,172,173]. It is noteworthy that it exhibits osteoconductive properties, rendering it effective for bone graft extension and highly compatible with soft tissues, which is advantageous for applications in orthopedic and dental implants. Furthermore, studies have demonstrated that the combination of HA with human platelet-rich plasma significantly enhances bone regeneration in critical-sized defects, thereby illustrating its substantial regenerative potential [174]. The bioactivity of HA enables effective bonding with surrounding tissue, thereby facilitating bone regeneration and rendering it an optimal coating material for metallic implants to ensure bioactive fixation [170]. Given its exceptional bioactivity, osteoconductivity, and biocompatibility, HA is an ideal candidate for applications in bone implantation and tissue engineering [175]. It has been widely employed in bone repair, augmentation, and as a filler in bone or dental procedures, thereby demonstrating its versatility in the field of bone regeneration. In a study conducted by Bernardo et al. (2022), three-dimensionally printed porous scaffolds were developed using fused deposition modeling (FDM) from polylactic acid/hydroxyapatite (PLA/HA) composites with a high ceramic content (exceeding 20% by weight) [176]. The objective was to create a material that would support the regeneration of bone defects. The PLA/HA scaffolds exhibited mechanical properties that were found to be compatible with those of trabecular bone. In vitro degradation tests demonstrated that HA neutralized the acidification resulting from PLA degradation while simultaneously releasing calcium and phosphate ions. Furthermore, PLA/HA scaffolds did not elicit immune responses in vitro, as evidenced by the absence of upregulation in activation markers or inflammatory cytokines in dendritic cells. Using human MSCs, it was observed that while pure PLA scaffolds exhibited osteoconductive effects, PLA/HA scaffolds markedly induced osteogenic differentiation even in the absence of classical stimuli. These findings suggest that 3D-printed PLA scaffolds with high HA concentrations hold promise for bone tissue engineering. While HA exhibits a number of beneficial characteristics, it is not without certain disadvantages. One significant drawback of HA is its poor biodegradability, which can impede the natural process of bone remodeling [177]. This represents a considerable challenge that highlights the necessity to develop more biodegradable alternatives, such as low-crystalline carbonated hydroxyapatite (L-CHA) [178]. Additionally, HA’s capacity to facilitate new bone formation is constrained, predominantly due to its elevated crystallinity and diminished specific surface area, which diminish its efficacy in promoting the attachment and proliferation of bone cells. Given these limitations, recent studies have demonstrated that the incorporation of various elements, including iron, strontium, cerium, and others, can enhance HA’s mechanical properties, as well as its antibacterial and corrosion resistance [179]. This composite approach renders HA a more promising candidate for applications in bone tissue engineering.

Ongoing clinical trials are focused on evaluating the safety and efficacy of calcium-based scaffolds, including β-TCP and HA, often in combination with MSCs or other biomaterials. The use of β-TCP, in combination with chitosan, is being explored as a bone substitute for mandibular fractures, with comparative analysis against autologous bone grafts. These trials primarily aim to assess the promotion of bone healing, infection rates, and functional recovery (NCT02081885) [180]. Additionally, β-TCP scaffolds seeded with MSCs are being investigated for the treatment of bone defects, with clinical and radiographic outcomes assessed over a period of 22 months (NCT02748343) [181]. Clinical trials involving HA include the evaluation of a starch–hydroxyapatite composite as a bone void filler for orthopedic and neurosurgical patients, with primary outcomes centered on material handling and long-term safety, assessed through radiographic follow-ups (NCT02910232) [182]. Furthermore, HA combined with MSCs is being studied for nonunion fractures, comparing its effectiveness to autografts (NCT01626625) [183]. Despite extensive research efforts, many of these trials have yet to yield conclusive results. These studies are crucial for determining the long-term viability of calcium-based bone substitutes in clinical applications.

## 9. Cell-Containing Scaffolds

Integrating cells with scaffolds represents a transformative approach in bone tissue engineering, with the potential to substantially improve regenerative outcomes. By pre-seeding scaffolds with cells such as mesenchymal stem cells or osteoprogenitor cells, the bioactivity and osteoinductive properties of these constructs are significantly enhanced. This cell–scaffold synergy fosters robust cellular attachment, proliferation, and differentiation, accelerating bone formation and improving implant integration.

Research in this area has yielded promising results. For instance, research in bone regeneration has highlighted significant advantages of using stem cells to enhance healing in critical-sized defects. Kalaiselvan et al.’s study demonstrated that Bone Marrow-Derived Mesenchymal Stem Cell-Laden Nanocomposite Scaffolds can significantly accelerate bone repair. The MSC-loaded scaffolds not only provide essential osteogenic cells but also release growth factors that create an ideal environment for bone regeneration [184]. Similarly, Kiany et al.’s work with collagen-poly(3-hydroxybutyrate)-carbon nanotubes scaffolds loaded with MSCs improved structural stability and demonstrated substantial bone formation, emphasizing the potential of cell-loaded scaffolds to reinforce scaffold integrity while promoting regeneration [185]. Additionally, Al-Qadhi et al.’s research explored the use of gingival mesenchymal stem cells (GMSCs) as an alternative to bone marrow stem cells, showing comparable efficacy in enhancing new bone formation. This finding is particularly significant as GMSCs are easier to harvest and have lower associated morbidity compared to BMSCs, making them a promising alternative for clinical applications [186]. The ability of cell-based approaches to deliver both osteogenic cells and bioactive molecules positions them as highly effective strategies for addressing the challenges of bone regeneration.

Numerous clinical trials have been conducted over the years to investigate the efficacy and safety of MSCs as adjunctive treatments for bone nonunion. Specifically, these studies have explored the direct injection of MSCs into nonunion sites or their application to bone matrices, utilizing both outpatient percutaneous procedures guided by fluoroscopy and surgical implantation for larger bone gaps (NCT01206179) [187]. Key outcomes of these trials include the assessment of callus formation, clinical union—defined by pain relief and mechanical stability—and the monitoring of adverse effects through radiographic imaging and follow-up evaluations conducted at regular intervals (NCT01429012; NCT01581892) [188,189]. Despite the significant body of research, there remains a notable lack of studies assessing cell-containing scaffolds. While some studies have investigated these approaches, such as the application of autologous bone marrow concentrate (BMC) cells seeded onto β-TCP in combination with angle-stable fixation for the treatment of proximal humeral fractures (NCT02153372) and the implantation of stem cells in a collagen-based 3D scaffolds (NCT01958502; NCT06103396) [190,191,192]. While the majority of clinical trials are completed, they either fail to provide results or only present preliminary findings. Consequently, it is often difficult to trace the development and final outcomes of these trials.

Despite the compelling potential of cell-containing scaffolds, several challenges limit their clinical application. Sourcing high-quality cells, such as MSCs, presents both logistical and financial challenges, and maintaining cell viability and functionality during manufacturing is crucial for success. Additionally, the regulatory pathway for cell-based therapies remains stringent and protracted, requiring rigorous safety and efficacy testing to ensure patient safety. From a manufacturing perspective, scaling up the production of cell-laden scaffolds is complex and costly, necessitating the development of efficient and cost-effective production methods. Standardization and quality control are essential to ensure consistent product performance and clinical reliability, which requires carefully developed manufacturing protocols and stringent quality assessments. Nonetheless, continued research and technological advancements are gradually addressing these barriers. As these innovations progress, cell-containing scaffolds hold substantial promise to redefine bone tissue engineering and regenerative medicine, marking a significant step toward transforming clinical approaches to bone regeneration.

## 10. Implant Microfabrication Technologies 

Although a number of techniques for bone regeneration have been developed, there is a continued effort to identify more effective forms of bone graft substitutes. It seems inevitable that in the near future, bone graft substitutes will either be gradually replaced or enhanced by more efficacious treatment modalities, such as autologous stem cells derived from bone marrow aspirates. The advancement of bone graft substitute engineering is concentrated on the creation of biomimetic scaffolds that not only facilitate cellular proliferation and bone tissue formation but also encourage ECM production and vascularization while simultaneously withstanding mechanical loads. One method of producing cellular scaffolds with potential for clinical application is the electrospinning technique [193]. In the electrospinning process, a viscous stream of polymer solution is ejected from a cone-shaped droplet and then subjected to stretching in an electric field. The evaporation of the solvent causes the stretched jet to solidify and form nanofibres, which are then collected on a grounded manifold in the form of a non-woven fabric [194]. The electrospinning process is a popular method due to its capacity to alter materials at the nanoscale and its ability to produce highly porous materials, which are among its few advantages over other techniques. At present, a considerable number of companies have successfully commercialized medical devices based on nanofibres. To illustrate, a few examples of such applications in regenerative medicine include stent coatings (Biotronik, Lake Oswego, OR, USA), blood vessel implants (Nicast, Leicester, UK), and a biodegradable dura mater substitute (Medprin, Guangzhou, China). The electrospinning process has been employed by numerous authors to create nonwovens from a range of natural and synthetic polymers. Materials produced via the electrospinning method exhibit high porosity and gas permeability, as well as a high surface-to-volume ratio. The scientific literature has demonstrated that cell scaffolds in the form of a two-dimensional non-woven fabric facilitate cell adhesion and proliferation [195]. It is of great importance to design biomaterials that can mimic the ECM in order to enhance the efficacy of various therapeutic approaches. Three-dimensional electrospun fiber technology, which can be injected in a minimally invasive manner, has the potential to significantly enhance regenerative therapies. Moreover, the distinctive characteristics of highly electrospun nanofibers enable them to replicate the natural ECM of bone tissue [196,197]. In this context, cell-based matrices represent a promising approach for bone regeneration, leveraging the properties of ECMs and stem cells to enhance bone repair and formation. MSCs are a frequently utilized component of bone tissue engineering due to their capacity to differentiate into osteoblasts, which are essential for bone formation. The combination of MSCs with ECMs has been demonstrated to significantly enhance osteogenesis [198]. ECM-based scaffolds provide a biomimetic environment that supports cell adhesion, proliferation, and differentiation. For example, scaffolds derived from the ECMs of MSCs or preosteoclasts have been demonstrated to enhance bone regeneration by promoting the osteogenic differentiation and migration of MSCs [199]. The integration of ECM with synthetic materials, such as polycaprolactone (PCL) or true bone ceramic (TBC), results in hybrid scaffolds that exhibit enhanced mechanical properties while maintaining their bioactivity. This enables effective support for cell proliferation and osteogenic differentiation [200]. Notably, nanofibers, such as carbon nanofibers (CNFs), contribute significantly to the mechanical reinforcement and elasticity of bone implants, rendering them particularly advantageous for load-bearing applications. For instance, the incorporation of CNFs into PCL and mineralized hydroxyapatite composites markedly enhances adhesion strength and elastic modulus—key attributes for weight-bearing implants [201]. Similarly, the addition of cellulose nanofibers to calcium phosphate silicate cement has been shown to improve its mechanical properties to a level comparable with that of trabecular bone [202]. Furthermore, the integration of bioactive proteins, such as BMP and IGFBP5, within ECM-based scaffolds enhances bone regeneration by stimulating osteoblast differentiation and activity [198,203]. Several in vivo studies have demonstrated the efficacy of ECM-based scaffolds in promoting bone regeneration. For example, ECM-decorated poly(lactic-co-glycolic acid) (PLGA) scaffolds have been shown to significantly increase bone volume and mineral density in calvarial defect models, while microcarrier DBM scaffolds have exhibited enhanced vascularization and bone formation compared to block DBM [199,204].

Notwithstanding these encouraging outcomes, several obstacles remain, chief among them being the limited availability of cells and the necessity for efficient vascularization. Addressing these challenges is essential, and the objective of future research is to optimize scaffold design. Recent advancements in additive manufacturing and microfabrication technologies, including 3D printing, 3D bioprinting, and other novel fabrication techniques, have facilitated significant progress in this area. These technologies enable the precise fabrication of scaffolds that closely replicate the structural and biological complexity of native bone tissue, thereby enhancing their potential for clinical application [205]. 3D printing allows for precise control over scaffold geometry, porosity, and interconnectivity. The fabrication process employs a range of materials, including biocompatible and biodegradable polymers such as PLA, PCL, and PLGA, osteoconductive ceramics such as HA and β-TCP, as well as 3D-printed titanium [206]. Hybrid composites incorporating these materials have demonstrated efficacy in achieving a balance between mechanical stability and biological functionality, making them suitable for applications in craniofacial reconstruction and orthopedic interventions. For example, scaffolds fabricated from PLA/HAp composites have shown the ability to support bone regeneration while maintaining mechanical properties compatible with trabecular bone [207]. Studies have demonstrated their capacity to support osteogenesis and vascularization in vivo, indicating their clinical utility [176].

Moreover, the clinical trial “Polycaprolactone/Tricalcium Phosphate (PCL/TCP) vs. Titanium Orbital Implant: Randomized Trial” evaluated the efficacy of 3D-printed PCL/TCP implants in orbital wall reconstruction compared to traditional titanium mesh implants. This study shows advancements in additive manufacturing and microfabrication technologies, particularly in the development of bone scaffolds and implants. The trial involved 80 participants aged 21 to 70, randomized into two groups receiving either PCL/TCP or titanium mesh implants. Assessments included diplopia, enophthalmos, visual acuity, globe mobility, and orbital contour symmetry, with CT scans performed immediately postoperatively and at 12 months. The use of PCL, a biodegradable polymer, combined with TCP, an osteoconductive ceramic, facilitated the creation of implants with optimized porosity and interconnectivity, promoting bone ingrowth and vascularization [208]. 3D printing also enables the production of implants with optimized porosity and complex lattice structures, enhancing biomechanical integration and osseointegration [209]. For example, titanium lumbar interbody cage implants incorporate advanced architectural designs, such as radiolucent lattice structures and features that facilitate bone graft packing and promote fusion. Porous titanium is often employed to approximate the modulus of elasticity of native bone, reducing implant subsidence and improving bone ingrowth [210]. These advancements not only improve clinical outcomes but also represent a shift toward tailored implant designs informed by patient-specific anatomical and pathological considerations such as reconstructing deformities after tumor excision [211]. 3D bioprinting further extends the capabilities of traditional 3D printing by incorporating bioinks—hydrogel-based materials containing living cells or bioactive molecules—enabling the production of cell-laden scaffolds that closely mimic the native extracellular matrix. Bioinks such as gelatin methacryloyl (GelMA), alginate, collagen, and fibrin have demonstrated their ability to support cell proliferation and differentiation [212]. Preclinical investigations have shown that 3D bioprinting enables the fabrication of patient-specific scaffolds capable of promoting healing in critical-sized bone defects [213]. Emerging technologies such as three-dimensional printing (3DP) add another dimension to personalized medicine by transforming patient imaging data into highly detailed, anatomically accurate 3D models. These models facilitate preoperative planning and the optimization of implant design [214]. For example, the use of 3DP has enabled the creation of patient-specific models for complex spine surgeries, improving surgical precision and advancing the development of anatomically compatible implants [215]. Although not yet in common clinical practice, this technology represents a significant step toward personalized medicine, enabling the development of therapeutic solutions tailored to individual patient profiles.

Further advancements in fabrication methodologies, including multiphoton lithography and laser-assisted bioprinting, have enabled the creation of complex microarchitectures with features such as microchannels for nutrient and oxygen transport and precise cell placement for tissue engineering applications. These techniques have shown particular utility in the fabrication of intricate scaffold architectures necessary for complex tissue regeneration [213,216].

The clinical implications of these innovations are significant. From 3D-printed scaffolds for craniofacial and maxillofacial reconstruction to fabricated spinal implants with optimized structural properties, these technologies address critical clinical needs. As 3D printing continues to evolve through the integration of artificial intelligence and advanced imaging technologies, the development of fully customized, patient-specific solutions is becoming increasingly feasible. These advancements hold the potential to redefine the future of orthopedic and reconstructive medicine, offering a pathway toward improved therapeutic outcomes and precision care.

## 11. Conclusions

Significant advancements in bone tissue engineering have led to the development of innovative materials and methods for bone regeneration. However, the search for the optimal bone substitute remains a critical objective in this field. Such a substitute must combine essential properties, including biocompatibility, bioresorbability, osteoconductivity, osteoinductivity, and adequate mechanical strength to support and stimulate the body’s natural bone regeneration processes. Achieving this balance ensures that the material can initially provide structural support while gradually being replaced by new bone tissue during the healing process. Despite progress with synthetic materials like ceramics, polymers, and composites, each class faces inherent limitations. Additionally, the incorporation of bioactive molecules, growth factors, and cellular therapies has shown promise in enhancing osteoinductive capabilities; however, achieving controlled and sustained release remains a substantial challenge. The search for an ideal substitute is further complicated by the need to tailor materials to specific clinical requirements and patient factors, such as age, underlying health conditions, and the defect’s location. Current research increasingly focuses on biomimetic approaches that mimic the natural architecture and biochemical environment of bone tissue. These strategies, including three-dimensional printing and bioactive coatings, hold the potential to bridge the gap between artificial implants and the complex regenerative capabilities of natural bone. Nonetheless, the long-term efficacy, safety, and cost-effectiveness of these approaches must be rigorously validated through preclinical and clinical trials. Furthermore, while long-term studies are ongoing for new and emerging implants and biomaterials to provide a clearer understanding of their sustained performance and clinical translation potential, more established bone implants, such as titanium-based implants, have demonstrated over 20 years of safe clinical use with a well-documented history of minimal adverse effects. Moreover, the growing field of personalized medicine, combined with the development of made-to-order implants, may represent the future of bone tissue engineering. By tailoring implants to the unique anatomical and biological needs of individual patients, this approach has the potential to significantly enhance both the functional outcomes and long-term success of bone regeneration strategies. In summary, while existing materials and techniques provide valuable tools for bone regeneration, they have yet to meet the stringent criteria required for broad clinical application. Advancing this field requires interdisciplinary collaboration across materials science, biology, and engineering to address the limitations of current bone substitutes. Success in this endeavor is essential to develop solutions that not only meet the diverse challenges of bone repair but also significantly improve the quality of life for patients with critical bone defects. Therefore, the pursuit of an optimal bone substitute remains a dynamic and evolving research area, continually driving innovation to enhance patient outcomes in orthopedic and maxillofacial applications.

## Figures and Tables

**Table 1:** Functional roles of selected peptides in bone healing and regeneration. (Based on: [95,97,98,101]).

Functional Category	Peptide	Role
Osteo-differentiation	PTH1-34 Recombinant form of N-terminally truncated and 34-amino acid-containing human parathyroid hormone (1–34)	Promotes osteoblast formation by activating the SIK2, Wnt4, and cAMP/PKA signaling pathways, thus enhancing osteogenesis and bone mineralization.
Facilitate cell adhesion	P-15 Osteogenic cell-binding peptide	Enhances bone healing through an “attract, attach, activate” mechanism, minimizing ectopic growth risk. Demonstrated high fusion rates in clinical studies.
Contribute to neovascularization	TP508 Peptide fragment of the receptor-binding domain of the native human thrombin molecule	Accelerates bone formation, facilitates healing in bone defects, and mitigates the adverse effects of fibrosis and complications associated with fractures.

**Table 2:** Comparison of calcium-based bone graft materials by commercial product examples, advantages, and disadvantages. (Based on: [54,152,153,154,155,156,157,158,159,160,161,162,163,164,165,166,167,168,169]).

Forms of Calcium-Based Bone Grafts	Product (Manufacturer)	Advantages	Disadvantages
Calcium phosphate cements (CPC)	- Norian™ SRS (DePuy, Warsaw, IN, USA) - ChronOS™ Inject (DePuy) - BoneSync™ (Arthrex, Naples, FL, USA) - Ossilix™ (Exactech, Gainesville, FL, USA) - HydroSet™ (Stryker, Singapore) - Quickset™ (Arthrex) - α-BSM (DePuy) - CopiOs^®^ (ZimVie Spine, Palm Beach Gardens, FL, USA) - Graftys^®^ (Graftys, Vaulx-en-Velin, France) - BIOPEX-R^®^ (HOYA Technosurgical Corporation, Tokyo, Japan)	- Exhibits a chemical composition closely resembling the mineral component of bone, which enhances its bioactivity and osteoconductivity, facilitating strong integration with host bone - Resorption rate can be adjusted by modifying the phase composition, allowing for tailored clinical applications - Demonstrates self-hardening capabilities through body-temperature-induced dissolution and precipitation reactions - Minimal shrinkage during the setting process - Ability to fill cavities of complex configuration - Absence of exothermic reaction during setting, preventing thermal damage to adjacent tissues - Injectable, reducing the invasiveness and risk of infection during the operation - Can be used as delivery systems for therapeutic peptides, antibacterials, anticancer drugs, anti-inflammatory drugs, or growth factors	- Brittle - Relatively low bending/flexural strengths - Poor mechanical properties that limit broader clinical application - Can only be used in combination with internal or external fixation or in low- or non-load-bearing applications - If not adequately supported, there is a risk of poor integration with existing bone, leading to potential graft failure - Potential for inflammatory reaction and embolism - Slow degradation rates, lower than new bone formation rate, which might limit natural healing - The injection and setting of CPCs can be technically challenging, requiring skilled application to ensure optimal outcomes - Intrinsic porosity reduces strength
Calcium sulfate (CaSO_4_)	- OsteoSet^®^ (Stryker) - MIIG^®^ INJECTABLE Graft (Wright Medical Technology, Arlington, TN, USA) - CERAMENT^®^ (Bonesupport, Lund, Sweden) - PRO-DENSE^®^ (Stryker) - Stimulan^®^ (Biocomposites, Biel, Switzerland) - BondBone^®^ (MIS Implants Ahihud, Israel) - PRO-STIM™ (Stryker)	- Degrades primarily through dissolution rather than cell-mediated resorption, making it suitable for specific applications such as filling small bone defects - Osteoconductive - Exhibits compressive strength greater than cancellous bone - Suitable for filling small bone defects or used with rigid internal fixation - Inexpensive and easy to prepare - Well-accepted by surrounding tissues, minimizing the risk of adverse reactions and facilitating smoother integration - The dissolution process acidifies the surrounding environment, which can enhance its antimicrobial effectiveness, making it a superior adjunct to non-osteogenic materials - Can act as a vehicle for drug delivery (antimicrobials, antibacterials, etc.)	- Provides no internal structural support - Absence of macroporosity, inhibiting osteoconduction within the material - Not suitable for large bone defects - Rapid biodegradability (biodegrades after 4–8 weeks, much faster than the calcium phosphate cements)—this rapid dissolution could be problematic, as it is faster than the ingrowth of new bone, and therefore, the void may not be filled throughout the process - Due to its rapid resorption and lack of structural support, calcium sulfate may not be suitable for load-bearing applications
Hydroxyapatite (HA)	- Pro Osteon (Biomet, Warsaw, IN, USA) - BoneSource™ (Osteogenics, Lubbock, TX, USA) - ReproBone^®^ (Ceramisys, England, UK) - Calcibon^®^ (Biomet) - PerOssal^®^ (Osartis, Münster, Germany)	- Strong stability and biological activity - Exhibits good cell affinity, which promotes adhesion and proliferation of the osteoblasts and direct bone integration - The most stable calcium phosphate with low solubility in physiological environments - Has a chemical structure highly analogous to that of natural bone minerals, promoting biocompatibility and integration with host tissue - Nontoxic, minimally inflammatory, showing no adverse immune reactions or irritation in vivo - Size and porosity of HA can be adjusted in order to increase the osteoconduction - Compared to autograft, HA blocks provided quicker fusion and superior stiffness - Can encourage the formation of new blood vessels, which is crucial for the healing and regeneration of bone - Can be engineered into various forms, such as granules, scaffolds, or coatings for implants, allowing for tailored applications in different surgical contexts - Can be combined with growth factors, cells, and/or molecules for better osteoinductivity	- Inelastic and brittle, which can lead to structural fractures and limitations in molding into complex or load-bearing geometries, potentially impacting its efficacy in bone regeneration applications - Limited fracture toughness, which restricts its use in high-stress environments, as it may compromise structural integrity under mechanical load - Poor mechanical properties, including low biodegradation and tensile strength - Exhibits a very slow resorption rate and is minimally absorbed by the body, which can impede the natural bone remodeling process. This prolonged presence may result in a mismatch between graft resorption and new bone formation, potentially affecting the healing dynamics in certain clinical applications - Low fatigue resistance in a physiological environment
Tricalcium phosphate (TCP)	- genex^®^ (Biocomposites) - Allogran-R^®^ (Biocomposites) - Cerasorb^®^ (Curasan, Kleinostheim, Germany) - chronOS™ (DePuy) - SynthoGraft^®^ (Bicon, Boston, MA, USA) - Vitoss^®^ (Stryker) - CELLPLEX^®^ (Wright Medical Technology) - MasterGraft^®^ (Medtronic)	- High similarity in structure and composition to bone mineral - Excellent biocompatibility - Osteoconductive properties - Alloplastic - Facilitates bone regeneration by providing a scaffold for the in-growth of cellular and vascular components - Degradation kinetics closely align with endogenous bone formation rates, facilitating a controlled replacement by new bone tissue as it resorbs	- Brittle, which limits its use in load-bearing applications due to low mechanical strength and fracture toughness - Lacks osteoinductivity and osteogenicity - Poor mechanical properties, which makes it unable to resist against fatigue and insufficient holding power, thereby making it susceptible to scaffold collapse or internal fracture, which restrains its application in weight-bearing areas

## Data Availability

Not applicable.
